# Identifying and Minimizing Errors in the Measurement of Early Childhood Development: Lessons Learned from the Cognitive Testing of the ECDI2030

**DOI:** 10.3390/ijerph182212181

**Published:** 2021-11-20

**Authors:** Claudia Cappa, Nicole Petrowski, Elga Filipa De Castro, Emily Geisen, Patricia LeBaron, Betania Allen-Leigh, Jean Marie Place, Paul J. Scanlon

**Affiliations:** 1 United Nations Children’s Fund, New York, NY 10017, USA; ccappa@unicef.org (C.C.); edecastro@unicef.org (E.F.D.C.); 2 Qualtrics, Raleigh, NC 27603, USA; egeisen@qualtrics.com; 3 RTI International, Research Triangle Park, NC 27709, USA; plebaron@rti.org; 4 Instituto Nacional de Salud Pública, Cuernavaca, Morelos 62100, Mexico; ballen@insp.mx; 5 Department of Nutrition and Health Science, Ball State University, Muncie, IN 47303, USA; jsplace@bsu.edu; 6 National Center for Health Statistics, Hyattsville, MD 20782, USA; wyv6@cdc.gov

**Keywords:** early childhood, cultural relevance, cognitive testing, questionnaire design

## Abstract

Challenges in measuring early childhood development (ECD) at scale have been documented, yet little is known about the specific difficulties related to questionnaire design and question interpretation. The purpose of this paper is to discuss the challenges of measuring ECD at scale in the context of household surveys and to show how to overcome them. The paper uses examples from the cognitive interviewing exercises that were conducted as part of the methodological work to develop a measure of ECD outcomes, the ECDI2030. It describes the methodological work carried out to inform the selection and improvement of question items and survey implementation tools as a fundamental step to reduce and mitigate systematic measurement error and improve data quality. The project consisted of a total of five rounds of testing, comprising 191 one-on-one, in-depth cognitive interviews across six countries (Bulgaria, India, Jamaica, Mexico, Uganda, and the USA). Qualitative data analysis methods were used to determine matches and mismatches between intention of items and false positives or false negative answers among subgroups of respondents. Key themes emerged that could potentially lead to systematic measurement error in population-based surveys on ECD: (1) willingness of child to perform task versus ability of child to perform task; (2) performing task versus performing task correctly; (3) identifying letters or numbers versus recognizing letters or numbers; (4) consistently performing task versus correctly performing task; (5) applicability of skills being asked versus observability of skills being asked; and (6) language production versus language comprehension. Through an iterative process of testing and subsequent revision, improvements were made to item wording, response options, and interviewer training instructions. Given the difficulties inherent in population-level data collection in the context of global monitoring, this study’s findings confirm the importance of cognitive testing as a crucial step in careful, culturally relevant, and sensitive questionnaire design and as a means to reduce response bias in cross-cultural contexts.

## 1. Introduction

The best practices for survey design are well established and have been documented [1]. However, research literature documenting the development of new survey instruments remains limited [2], and reports about the pre-testing of survey tools focus predominantly on psychometric validity [3].

Field testing of survey items is an essential step in establishing psychometric validity, question quality, item performance, reliability, and addressing important sources of measurement error. This said, field testing alone does not provide sufficient information on all aspects of questionnaire design, including respondent misinterpretation of questions and response options.

Measurement error undermines the intent of survey items, which is to adequately capture underlying theoretical or conceptual constructs. Reducing and mitigating systematic measurement error, including problems with question wording and translation, response options, and sequencing and presentation of items, is thus crucial in the development of data collection instruments.

Chief among the difficulties with developing population-level measures is establishing measurement invariance that includes comparing the applicability of constructs, and items to measure them, across a range of different groups within and across populations and contexts. Establishing measurement invariance is paramount but difficult to achieve when working across various cultures, languages, and education levels of respondents who may understand constructs differently.

Previous studies have shown that cultural adequacy and cross-cultural comparability are two main challenges of early childhood development (ECD) measurement [4]. Major issues in ECD measurement for the purpose of global monitoring include misalignment of items with local expectations of children’s development and questions that reflect an association with family wealth and certain environmental exposures rather than capturing the acquisition of specific milestones. Additionally, issues with the response process involving interpretive errors, recall inaccuracy, and poor comprehension that limit cross-cultural validity have also been documented [5]. Measurement error can be particularly serious when it correlates with specific respondent attributes (for example, respondent’s level of education). This type of systematic measurement error can generate important biases that compromise data quality, which can lead to wrongful conclusions being taken from the data. Response biases can be minimized by careful, culturally sensitive questionnaire design and testing of question items using both quantitative and qualitative approaches.

Nearly two decades ago, the United Nations Children’s Fund (UNICEF) pioneered one of the first attempts to collect population-level data on ECD, through the Early Childhood Development Index (ECDI), which has been implemented in more than 70 countries (mostly through its inclusion in the Multiple Indicator Cluster Surveys or MICS) [6]. With the adoption of the Sustainable Development Goals (SDGs) in 2015, UNICEF, in collaboration with key partners, initiated a process to revise the ECDI and develop a new population-level instrument to measure the proportion of children aged 24 to 59 months who are developmentally on-track in the domains of health, learning, and psychosocial well-being. With this in mind, the ECDI2030 was developed. This new measure comprises 20 questions administered to mothers or primary caregivers that ask about the way their children behave in certain everyday situations and the skills and knowledge they have acquired. The ECDI2030 generates data that can be used for monitoring and reporting on SDG indicator 4.2.1.

Testing of the ECDI2030 followed a mixed methods approach, aimed at addressing the psychometric validity of the measure, its suitability to be implemented at scale in national household surveys, and its relevance to capture childhood development across different contexts. This latter aim was assessed through cognitive interviewing.

Cognitive interviewing is a standardized qualitative methodology whose purpose is to evaluate survey questionnaires in terms of comprehension and interpretation with the goal of enhancing cross-cultural appropriateness of survey items, reducing response bias, and minimizing measurement errors [7]. The question response process [8] is the underlying theory that guides cognitive interviewing. Individuals typically interpret survey questions through a four-step process: (1) they first comprehend the underlying construct; (2) then they recall the information needed; (3) they judge their answer; and finally (4) they map their answer onto one of the available response categories. The primary benefit of cognitive interviewing over non-qualitative evaluation methods is that it provides rich, contextual data on how respondents move through the four steps, apply lived experiences, and formulate responses to survey items based on interpretations, experiences, and specific cultural backgrounds [7,9,10]. Thus, cognitive interviewing data allow researchers and survey designers to understand whether or not a question and its response options are capturing the original intent and meaning of the questions, as well as identify problematic phrasing/wording of items that lead to false positive or false negative answers. This is crucial in population-level ECD measurement due to the need for the score to be accurate in depicting children’s developmental status as reported by mothers or primary caregivers, and therefore relevant to inform policies and programs.

The purpose of this paper is to discuss the challenges of measuring ECD at scale in the context of household surveys and to show how to overcome them. The paper uses examples from the cognitive interviewing exercises that were conducted as part of the methodological work to develop the ECDI2030. Given the complex, multi-dimensional, and culturally bound nature of ECD, this paper aims to contribute to the body of evidence on ECD measurement by illustrating the role of cognitive testing to reduce measurement error for population-level ECD monitoring.

## 2. Methods

A total of 63 questions were selected for testing. These included the 10 questions from the original MICS ECDI, together with an additional 53 questions that resulted from a comprehensive desk review of more than 500 items from 11 existing instruments that measure child development. Items in this inventory were first categorized into one of the three domains comprising SDG indicator 4.2.1 (i.e., health, learning and psychosocial well-being). Within each of these domains, items were then further grouped into the subdomains from the conceptual framework that had been developed through an extensive review of research literature and existing evidence on ECD. Technical experts in the field of ECD measurement reviewed and shortlisted items in the inventory on the basis of the following criteria: policy relevance, intervenable/actionable, cross-cultural applicability, pragmatic/easy to administer, accurate reporting by caregivers, and being known to have strong empirical evidence/predictive validity. A minimum of two experts had to recommend the item in order for it to be retained as part of the draft set.

The original MICS ECDI underwent cognitive testing in India and Jamaica to understand which items should be dropped and which should be retained for inclusion in the set of questions to be further tested. From this initial testing, two items were dropped due to important issues in item interpretability [11].

The remaining 61 items (Supplementary Table S1) were then evaluated using iterative cognitive interviews following the typical procedure [10,12,13], wherein the results from each round led to revisions that were then evaluated in future rounds. The 61-item questionnaire was first evaluated in the United States with a total of 30 interviews. After this round, 19 questions were modified, one was removed, and four were newly added. Next, in Mexico and Bulgaria, both the original and revised questions were evaluated across 47 and 30 interviews, respectively. The analysis of these findings resulted in revisions to nine questions. Finally, 39 interviews were conducted in Uganda, resulting in 28 revised questions. The fifth and last round of testing occurred again in Mexico on a subset of 22 items that continued to have interpretation issues. These interviews were carried out with mothers of 32 children as part of a dedicated field test of the ECDI2030.

While there were different implementing teams in each country (RTI International in the USA, the Institute for Public Policies Studies in Bulgaria, the National Institute of Public Health of Mexico, and Uganda Bureau of Statistics), all interviewers received standard training from the same set of core trainers. Additionally, a standard protocol for conducting the interviews was followed in all rounds. These steps were taken in order to replicate standard conditions for survey implementation and to ensure that different implementing partners complied with the same set of standard procedures.

### 2.1. Sample Specification and Participant Recruitment

Within each site, a purposive sample was constructed to maximize diversity across a number of respondent characteristics including child age, child sex, household location, levels of caregiver education, and child disability status. Additionally, in Mexico mothers of indigenous ethnicity were specifically recruited. To be eligible, respondents had to be the primary caregivers of at least one two- to four-year-old child.

Local staff from the implementing partner in each country used a variety of recruitment procedures to identify potential participants. General strategies to identify potential participants included working with daycare centers, preschools, or community or state agencies that interact with mothers of young children with and without disabilities. Additional recruitment strategies varied by country. For example, in India a school for children with disabilities used its network to contact potential respondents. In the United States, online community message boards were used that included a link to a screener to determine eligibility. In Jamaica, Mexico, and Bulgaria, staff worked through word-of-mouth invitations and snowball sampling to recruit mothers who were neighbors or acquaintances. In Uganda, staff from the local implementing agency worked with chairmen (local leaders) from sampled areas to identify participants with desired attributes prior to the data collection.

During recruitment, staff at each site contacted eligible participants to answer any questions that they had and to schedule the date, time, and location of interviews. Staff also recruited additional individuals for a wait list in case those scheduled canceled or missed their interview appointment. Demographic characteristics of the final sample of participants are described by country in Table 1.

### 2.2. Interviewer Training

Training for each round included a two-day, in-country, interactive training to prepare respective staff to recruit participants and conduct the interviews. The training focused on the fundamentals of cognitive interviewing, reviewing the interview guide, and familiarizing staff with project procedures and logistics. Interviewers reviewed the items about early childhood development, the descriptions of each item, probes in the interview guides, procedures for consent and note-taking, as well as the schedule for interviews. A selection of interviews during the first day of fieldwork was observed by trainers, either in person or using video conferencing with a camera focused exclusively on the interviewer (without recording the image), with suggestions and corrections given after each interview that was observed.

### 2.3. Interview Methodology

Intensive concurrent verbal probing was used during the interviews to collect data about the response process. Participants were asked to listen to each question as the interviewer read it aloud and then provide a response. Following this question-response process, participants were then asked to talk through what they had thought about as they answered each question. Follow-up probes consisted of general probes (e.g., “In your own words, what is this question asking?”, “Tell me how you came up with your response?”) and question-specific ones, such as probes about terms or concepts that might have been confusing (e.g., “What does ‘identify’ mean as it was used in this question?”, “What kind of objects were you thinking of?”). These targeted probes attempted to ascertain exactly which constructs the participants were considering, how they were judging and formulating their response, and whether participants comprehended the constructs. Finally, spontaneous *ad-hoc* probes explored inconsistencies in reporting and topics.

Questions and probes were written in English and then translated into Hindi, Marathi, Bulgarian, and Spanish by professional translators. The translation of the items was discussed and edited when needed with bilingual interviewers during the first day of training.

### 2.4. Data Collection

Interviews were conducted throughout 2016 and 2017. Interviews in Jamaica, the United States, and Uganda took place in English. Interviews in India were conducted in English, Hindi, and Marathi. Interviews in Bulgaria and Mexico took place in Bulgarian and Spanish, respectively. Interviews were conducted face-to-face in the offices of the local implementing partner (Jamaica, United States, Mexico, and Bulgaria), a local school (India), as well as at participants’ homes (United States, Mexico, and Uganda). Interviewers reviewed a consent form with each participant prior to audio recording the interview. The research protocols for India and Jamaica were reviewed by a government Ethics Review Board; the overall research protocol and consent forms for the later rounds were reviewed by a centralized Institutional Review Board. In the case of Mexico, additional ethical approval was sought from the local institutional ethics and research committees. Each country used a consent form that described the purpose of the research, informed participants that their answers were confidential and voluntary, and described the incentive for the research.

Interviewers collected demographic data, including whether the child had any formal schooling, the socioeconomic status of the family, education level of the parent, marital and employment status, language spoken in the household, and number of children living in the household. Interviewers took notes throughout the interviews, documenting any misunderstandings or items that were difficult for participants by noting participants’ answers to each of the items, responses to probes, and other notes. At the conclusion of each interview, they also compiled the notes into an electronic template for analysis. Interviews typically lasted 60–75 minutes and participants received small monetary (in India, Jamaica, and the United States) and non-monetary (in Mexico, Bulgaria, and Uganda) incentives in appreciation for their time.

### 2.5. Analysis

The analysis of cognitive interviewing data involves an iterative synthesis and reduction of the findings. During each iteration, data were reduced such that meaningful content was systematically extracted to produce a summary detailing each question’s performance.

Data analyses in India and Jamaica were conducted using the Q-Notes software. Q-Notes is a qualitative analysis software, developed by the National Center for Health Statistics and specifically designed for cognitive interviewing in large, multi-site projects [14]. In the United States, Mexico, Bulgaria, and Uganda, the research teams conducted analyses using Excel. Audio recordings were accessed to review interviews, as needed.

Information gathered during probing was compared to item descriptions to determine matches and mismatches between the intention of the item and participants’ answer and interpretation of the item. Analysis of the information followed a stepped approach consisting of (1) reading through the item description; (2) reviewing the responses to each item; (3) reviewing contradictions and answers that changed from yes to no, or from no to yes, upon probing; (4) describing interviewee comments on how they arrived at their answer; and (5) coding answers according to mismatches resulting in either false positives or false negatives for the item, and registering other issues with item interpretation.

The information was examined in terms of underlying patterns related to the child’s age and other mother and child demographics. Such results provided insight into whether the item might or might not be appropriate for a given characteristic or age group. The analysis also aimed to reveal themes among items that consistently used the same terms or measured similar constructs. These themes, such as the performance of items that measure a child’s ability to engage in a behavior as opposed to their willingness, were included in the results. Finally, the use of examples was reviewed to illustrate how examples influenced response patterns.

## 3. Results

Results are presented in three sections. First, results are presented according to the four stages of the cognitive response process model (i.e., comprehension, retrieval, judgment, and response) to identify potential areas of misinterpretation in the ECDI2030 questions. Second, results from maternal and child subgroup analyses are outlined with potential interpretative differences based on maternal education, as well as language and cultural background. Finally, key changes that informed the final set of the ECDI2030 items are reported.

### 3.1. Main Issues in the Cognitive Response Process Model

Analysis of cognitive testing results, mapped onto the four stages of the cognitive response process model, revealed potential areas of response bias and systematic measurement error.

*Comprehension*. Comprehension refers to issues that participants may experience in understanding concepts or terms within the question, leading to vague interpretations of the question’s central concept. We identified five main issues related to item interpretation.

The first pertains to confusion over concepts and terms used in the question, and respondents’ abilities to distinguish between concepts. For instance, how respondents interpreted and understood the terms “identifying”, “recognizing”, and “knowing” in items that asked about letters, numbers, and colours did not appear to consistently capture the same action across respondents. This issue is described further in Section 3.2 Significant Subgroup Findings.

Questions about children’s abilities to complete tasks led to a second set of comprehension issues. When reporting whether children could undertake an activity (e.g., going to the bathroom alone, or doing something that a familiar adult asks), many respondents considered both their children’s willingness and ability to do so. However, the analytic interest of these items was only with a child’s ability to perform the task.

The third set of issues stemmed from confusion between a child performing tasks vs. performing them correctly. Participants tended to base their responses on the fact that their child could complete an activity (e.g., counting 10 objects, using pronouns), even if he/she could not complete it correctly. However, completing an activity *correctly* is the intended construct in these items. To mitigate this in later rounds, the term ‘correctly’ was added either to the question text or the instructions for interviewers to indicate that the items seek to measure whether children can complete these tasks correctly. These changes limited reports of attempting tasks incorrectly and thus appeared to focus respondents’ interpretations (see items 8 and 14 in Supplementary Table S2). Similarly, in an item that asked whether children can consistently state an object’s name, participants were unclear of the meaning of ‘consistently,’ often conflating it with ‘correctly,’ even when the intent of the question does not require that the child use the standard name or pronounce it correctly, only that the child always uses the same word for the object. This item was revised to include a definition of consistently and retained for inclusion in the ECDI2030 (see item 9, Supplementary Table S2).

In some cases, comprehension issues emerged with respect to certain words that had more than one meaning in some languages but not others. For instance, with the question, “Does (name) get distracted easily?”, the word “distraction” has two meanings in Spanish. One meaning is similar to the meaning of the word in English and the other meaning refers to whether a child is attracted to or interested in a variety of things. As a result, some Spanish-speaking mothers interpreted the question differently than intended. The language issue in this item could not be mitigated and the item was thus eventually dropped from inclusion in the ECDI2030.

Lastly, the interpretation of the core construct was sometimes impacted by the use of examples. Many questions included examples of the concepts being asked about (e.g., “Can (name) talk about things that have happened in the past using correct language, for example, ‘Yesterday I played with my friend’ or ‘I ate an apple this morning’?) In these types of questions, participants had a hard time following the intent of the question and tended to focus on the specific examples listed. For example, with the question on talking about things in the past tense using correct language, many parents answered ‘No’ since their children did not understand the concept of “yesterday” correctly, could not use the past tense of irregular verbs (to eat), or could not say sentences as complex as the examples provided. During probing, however, parents revealed that their child could use the past tense correctly by saying things such as “I played.” In this item, the examples were intended to serve as a heuristic for understanding the item’s intent, but instead may have introduced measurement error. This item was dropped from inclusion in the ECDI2030.

*Retrieval.* Retrieval is the process through which a respondent searches his or her memory for the information needed to comprehend or answer the question. Errors related to retrieval include whether or not a participant has ever formed an attitude about the topic, whether they have the necessary knowledge to answer the question, whether the respondent has ever observed the behavior being asked, and whether the long-term memory mental calculations are too great.

In the testing of the ECDI2030 items, a common issue related to retrieval was when a participant did not directly observe the child’s behavior or the child completing a certain task, but rather assumed that the child was able to perform the task or demonstrate the behavior. For example, one question asked about whether the child can pick up a small object with two fingers, such as a stick or a rock from the ground. Although the intent of this item was clear to most participants, some participants indicated they could not recall they had seen their child do this, likely since picking up something in this way was not salient to the parent, which also touches on problems with comprehension. This item was eventually dropped from inclusion in the ECDI2030. Questions that assessed how the child reacts to seeing someone crying or on being aware of others’ preferences were excluded as well, since participants reported these scenarios had not occurred in the respondent’s presence or parents had never encountered this situation in the household.

Other questions were problematic at the retrieval stage since respondents were not familiar with some of the objects referenced in the question. For example, with the question, “Does (name) know that an elephant weighs more than a mouse?” some participants noted that their child had never seen an elephant before. This challenge may be specific to country contexts; children in certain countries may be less aware of animals that they have not encountered in-person. Similarly, children who have less access to picture books or media may only become aware of animals through in-person interactions. This item was dropped from inclusion in the ECDI2030.

*Judgement.* Judgement refers to deciding what is relevant to answer the question, whether the information requested is too sensitive, or what behaviors “count” towards the purpose of the question. Issues of relevance and applicability can lead respondents to report based on their perceptions or suppositions, rather than based on observations. For example, questions on whether the child could grab things with a finger and a thumb. Parents had not observed this behavior but assumed their child could do it since they had also not observed them failing to do it, so they answered “Yes”.

Another concern related to judgement has to do with participants varying in what they believe “counts” as a ”Yes” response to the question. For example, when asked if their child can sing a short song or repeat parts of a rhyme from memory by him/herself, some participants thought their child needed to be able to sing an entire song to answer “Yes,” while other parents thought just being able to sing part of the song should “count” for this question. Similarly, participants were not sure how exact the imitation should be of their child drawing a straight line in order to respond affirmatively. Such items were eventually dropped from the item set due to the challenge in making sure the respondent’s answers were based in harmonized or equivalent judgements.

Examples can influence participants to respond too specifically to the example and thus produce false positive answers. For example, when asked whether their child can follow two-step directions, the example “Go to the kitchen and get a spoon” may have led to false positives since respondents limited their interpretation to that specific action (going to get a spoon). However, since a spoon is typically kept in the kitchen, it was not clear whether respondents were answering “Yes” since their child could typically follow two-step directions or simply since their child could get a spoon. This item was eventually dropped.

*Response.* Response refers to the clarity of response options, as well as issues with how the answer is mapped onto available response options. Some questions were problematic since the questions provided only yes or no response options, but respondents wanted to provide an answer of sometimes. For example, caregivers in India and Jamaica were asked about whether or not their child was ever too sick to play, and were given only “Yes” or “No” as response options. A number of parents explained that their child was occasionally sick with the flu or colds, and obviously could not play then, but felt that the “Yes” answer category indicated that their child was sickly or chronically ill; they therefore answered “no” or refused an answer, feeling as though the answer categories did not map to their lived experience. Similarly, with the question “Does (name) become extremely withdrawn or shy in new situations?” a response scale with frequency options (e.g., never, rarely, sometimes, often, always) as opposed to a yes or no response option would more accurately allow participants to represent their children’s behavior overall. These items were excluded from inclusion in the ECDI2030 due to expressed problems in the response options. When asked if the child often kicks, bites, or hits other children or adults, participants wanted to qualify their ‘yes’ or ‘no’ responses with the frequency or recency of the behavior, often noting “not often,” “not constantly,” or “not anymore.” This item was retained in the ECDI2030 after changes to the frequency scale and including mention of a reference group for comparison.

Additionally, the word ‘often’ had varying interpretations among participants, with some using the term to indicate several times a week, whereas others understood the term to mean every time or consistently. In some cases, this issue was addressed by including scaled response options. In other cases, specific instructions were provided to interviewers, such as repeating the question and conducting probing, when they were unsure how to code the answer or if the respondent answers by saying ‘sometimes’ or ‘it depends’ on questions with only a yes/no response option.

### 3.2. Significant Subgroup Findings

*Influence of respondent education level.* As is common in cognitive evaluations of survey questions [10], an interaction between education level and question response was observed, where differences of interpretation emerged across participants with different levels of formal education. This was notable across the interviews conducted in Mexico and Bulgaria (respondents’ education was not collected in the testing carried out in Jamaica and India). These differences presented themselves in a couple of ways.

First, participants with a primary education or less tended to have more difficulty understanding some questions. For example, when asked if their child could read simple words, those with little formal education, some of whom were illiterate, could not distinguish whether the question was asking about identifying something in writing and saying it out loud, knowing letters, speaking with clear pronunciation, or saying phrases. Similar confusion occurred when asking about whether a child can identify numbers. There was a lack of clarity on whether the question was asking about seeing and saying numbers, saying and writing the numbers, or counting. Some respondents, when presented with these terms, comprehended the questions to be asking specifically whether or not their child knew and could say the name or identifier of the letter, number, or colour (i.e., knowing and saying the letter “a,” the word “one,” or the colour “red”). Other respondents interpreted these questions to be asking whether or not their child could point to or indicate letters, numbers, and colours when asked. Familiarity with some specific terms existed across education groups. For example, respondents with both high and low education in Mexico appeared to have an easier time understanding the term “recognize” (reconocer) over the term “identify” (identificar) within the context of the survey. The term ‘recognize’ best captured the intended behavior for items and these items were revised and retained.

*Influence of language and cultural background*. Translation is of vital importance in the design and evaluation of multinational, multiregional, and multicultural (3MC) surveys [15,16]. As expected for a survey initially designed in English, throughout the evaluation of the ECDI2030, the comprehension of some items was impacted by translation issues. For example, in Mexico, some questions were excessively wordy and therefore difficult to understand, even for mothers with college educations. For instance, the question “Can (name) easily switch back and forth between activities such as going back to a game or playing with a toy after being interrupted?” was too lengthy to easily understand for almost all mothers and was eventually dropped.

Translation issues can also interfere with the objective of the question. For example, in the question “Does (name) settle down after periods of exciting activity?” proved to lead to confusion since the word “excitement” in some of the languages used during the testing conveyed both positive and negative emotions, but the goal of the question was to focus only on positive emotions. This item was eventually dropped since this language issue could not be resolved.

Beyond the issue of translation, question response can also be influenced by cultural expectations. In Uganda for example, there was some evidence that respondents based some of their answers on cultural expectations. For instance, when asked if the child stops at least briefly when told no, one participant responded, “She has to stop because she must obey. I am her mother.” This mother, as well as other respondents, did not appear to strictly base their responses on observations, but instead of what was expected of their child within their cultural context. This item was dropped from inclusion in the ECDI2030 since participants thought about these scenarios from specific cultural perspectives and sought to elaborate on their response in ways that did not allow for a definitive ‘yes’ or ‘no’.

In addition to the impact of cultural expectations, local customs for education and childhood development also influenced responses to questions. Traditionally in Bulgaria, children are not encouraged to learn the alphabet or to read at such a young age. Participants explicitly made the distinction between reading and recognizing words, commenting that they would not expect children to be able to read at the age of four. This was also common among parents of younger children. For instance, one Indian mother noted that her two-year-old could not yet read any simple words, but that she was not concerned since she thought it was too early for that.

Providing examples in questions can help to illustrate a concept further. However, results of the cognitive testing indicated that not all examples were culturally appropriate across countries. One question asked whether a child usually finished an activity that he or she enjoys, such as doing a puzzle or looking at a book. In this case, the examples in the question assume which types of activities a child engages in.

### 3.3. Changes to Items

As noted above, the analysis of the cognitive interviews revealed potential for measurement error across all four phases of the question response process. These potential errors were not randomly distributed and were concentrated within certain types of participants and subjects. These findings led to a number of question wording and response scale changes, as well as changes to interviewer training instructions and other implementation materials. These revisions were made to improve the final items in the ECDI2030, and are presented in Supplementary Table S2.

*Item wording*. As cognitive interviews indicated that long and complex question wording was difficult for many participants to understand, some items were revised in order to shorten and simplify them. In other cases, wording was edited to better emphasize the purpose of the question. This included replacing words with more colloquial language, adding clarifying words, or removing words. For example, the word “correctly” was removed from item 11 to clarify the fact that the intent of the question was about fine motor skills as opposed to correctly writing names or letters. In other questions, examples were added to help convey the correct interpretation. For example, in item 15, the examples of colouring or playing with building blocks was added as types of activities children might do independently.

Additionally, some items were revised after the cognitive interviews revealed that participants’ interpretations varied or words were not consistently understood. For example, in item 12, the word “written” was deleted in the question “Can (name) identify all written numbers from 1 to 5?” This was carried out in order to avoid confusion about referring to written numbers (e.g., ‘five’) which also requires that a child be able to read versus identifying number symbols (e.g., ‘5’).

Finally, in many cases, editorial changes were made to improve ease of administration. These changes include subject/verb agreement (item 11 for example), insertion of the child’s name (item 2 for example), and integrating examples directly into the phrasing of the question as opposed to including them in parentheses (items 5, 6, 7, 8, 9, 13, 14, and 16).

*Response scales*. Analysis of the cognitive interviews suggested that participants’ responses would better reflect their lived experience if answer categories for some questions had wider ranges of possible answers-such as those with frequency scales that offered options of “Sometimes,” “Often,” or “Never”—as compared to binary “Yes” or “No” sets of categories. As a result, scaled response options were provided and the item stem was modified to add a reference to other children in order to streamline variability in responses based on age (item 20). In other cases, response options were changed to focus directly on frequency with answers including daily, weekly, monthly, a few times a year, or never (item 19). Providing a reference period of days, weeks, months, etc. was favored since it was perceived to lower participant burden and reduce false positives and negatives.

*Interviewer training instructions and other implementation materials.* Problems with information retrieval were addressed in the final ECDI2030 questions by either selecting examples that refer to aspects that can be easily observed in all contexts, or by allowing examples to be customized at country-level so that they are context relevant. For example, for the question “Can (name) do an activity, such as colouring or playing with building blocks, without repeatedly asking for help or giving up too quickly?” a specific instruction was included to make the examples context-relevant during translation and country-level customization of the ECDI2030. The instruction suggests that the text underlined in the question may be replaced if colouring or playing with building blocks are not typical activities for children in the country setting. The example should be replaced by similar activities that either are task-oriented (such as working on a puzzle or putting away clothes) or creative in nature (such as drawing, painting, or playing pretend games).

## 4. Discussion

The completion of 191 cognitive interviews across five rounds of testing in six different countries resulted in a set of items intended to measure ECD in household surveys. Information was gathered using intensive probing from mothers and caregivers of 2, 3, and 4 years old from diverse socio-economic and cultural backgrounds. Respondents’ interpretation of the items was compared to item descriptions to determine matches and mismatches between the intention of the item and participants’ answers. The results revealed issues with comprehension, definitions and use of examples throughout the questionnaire.

Key thematic areas emerged from the analysis that can could potentially lead to systematic measurement error in population-based surveys for early childhood development. They relate to the following terms and constructs: (1) willingness of child to perform task versus ability of child to perform task; (2) performing task versus performing task correctly; (3) identifying letters or numbers versus recognizing letters or numbers; (4) consistently performing task versus correctly performing task; (5) applicability of skills being asked versus observability of skills being asked; and (6) language production versus language comprehension. Results from the subgroup analysis suggested there are potential interpretive differences depending on maternal and child characteristics, including maternal educational attainment and language and cultural background.

The results shed light on some important considerations for reducing systematic measurement error of early childhood development items.

One important consideration is that items need to be understood by participants of all educational levels across cultural contexts. In some cases, including an example would be a way to make the question clearer, especially for women with less formal education. Examples can help allay measurement error by clarifying question intent and encouraging respondents to think of relevant examples from their own experiences which can help them answer the question accurately.

The final ECDI2030 set of questions is intended to be applied to all children two to four years old, and the questions were intentionally selected to reflect the increasing difficulty of the skills children acquire as they get older. Therefore, some questions might seem too easy or too difficult for some respondents, which might lead to frustration. Attention should be given as to how best to administer items to respondents with nonverbal children and fieldwork training should emphasize the importance of adequate handling of potential respondent questions or reactions in relation to this.

Interviewer instructions and training should also address how to handle a range of possible answers by the use of adequate probing that tackles the diversity of experiences respondents might have. Doing so may reduce respondent frustration and help identify the correct response coding. Interviewer instructions should be clear and precise in a way that can accommodate behaviors that manifest themselves most, but not all of the time, or behaviors that do not need to be demonstrated every single time to be indicative of something. Similarly, instructions on how to handle response options that reflect situations where the mother’s observations are based on partial exposure are also important and should be clearly conveyed during training.

Some participants in our sample indicated that an event did not usually happen (e.g., meeting new adults) or that the child had no opportunity to do something (e.g., helping others). These answers should be coded under a response category for “don’t know” and enumerators should be carefully trained on the use of this response option. The addition of this response option would help to address the issue of whether the skill being assessed has not been observed or is simply not applicable.

The ability of respondents to provide a response is contingent on the clarity of item translation. Items that are written in a way that are awkward, long, or hard to understand undermines interviewer ease of administration and, more importantly, respondent accuracy, even for mothers with college education. Prior to cognitive testing, time should be allotted for testing items’ clarity and flow. Although translations can be correct in terms of content, testing can help make sure each item is easily understood in terms of length and clause construction. This type of testing can be carried out by recruiting others to read the questions out loud and reporting on how easy or difficult it is to understand the question.

The limitations of this study are common to cognitive interviewing studies in general [10]. The standardized format of data collection (i.e., notes being inputted into an electronic template) helped to streamline and make visible the analytic process, which mitigated some concerns with data quality.

## 5. Conclusions

Cognitive testing is a fundamental step to inform item selection and improve data quality. Such testing should not be thought of as ancillary, but rather as a core component in survey design processes.

Given how complex and culturally driven ECD is, cognitive testing in this field has a heightened importance. Previously documented problems with ECD measurement, such as items that fail to be appropriate, reliable, and valid across multiple contexts, cultures, and different ethnic and socio-economic backgrounds, can be remedied by rewording questions, modifying response scales, eliminating unsuitable questions, identifying issues to be addressed at training, and improving interviewer techniques and data collection protocols. Practical, actionable steps can be taken to aid in question comprehension with specific implications for item wording improvement, response option development, interviewer instructions, training guidelines, and translation processes.

## Figures and Tables

**Table 1:** Socio-demographic characteristics of respondents from Jamaica, India, the United States, Mexico, Bulgaria, and Uganda.

Socio-Demographic Characteristics	Jamaica (*n* = 20)		India (*n* = 25)		United States (*n* = 30)		Mexico (*n* = 47)		Bulgaria (*n* = 30)		Uganda (*n* = 39)	
	*n*	%	*n*	%	*n*	%	*n*	%	*n*	%	*n*	%
**Child age**												
2 years-old	4	20.0	3	12.0	11	36.7	18	38.3	10	33.3	19	48.7
3 years-old	8	40.0	6	24.0	10	33.3	12	25.5	10	33.3	9	23.1
4 years-old	8	40.0	16*	64.0	9	30	17	36.2	10	33.3	11	28.2
**Child sex**												
Boy	13	65.0	15	60.0	NR	NR	28	59.6	NR	NR	NR	NR
Girl	7	35.0	10	40.0	NR	NR	19	40.4	NR	NR	NR	NR
**Disability status**												
Yes	0	0.0	10	40.0	2	6.7	11	23.4	7	23.3	3	7.7
No	20	20.0	15	60.0	28	93.3	36	76.6	23	76.7	36	92.3
**Indigenous or ethnic groups**												
Indigenous	NA	NA	NA	NA	NA	NA	4	8.5%	NA	NA	NA	NA
Roma	NA	NA	NA	NA	NA	NA	NA	NA	6	20	NA	NA
**Maternal educational level**												
Less than Secondary education	NR	NR	NR	NR	1	3.3	5	10.6	3	10	NR	NR
Secondary education and above	NR	NR	NR	NR	29	96.7	42	89.4	27	90	NR	NR

Note: In India, local researchers made the decision to include parents of five-year old children in the evaluation of the ECDI items. These nine respondents are included in the “four years old line” in this table. The data for Mexico do not include the additional interviews carried out with 32 mothers as part of the dedicated field test of the ECDI2030. NR, not reported; NA, not applicable.

## Data Availability

The data are not publicly available due to privacy and confidentiality issues.

## Supplementary Materials

The following are available online at https://www.mdpi.com/article/10.3390/ijerph182212181/s1, Table S1: Questions used for cognitive testing, by domain and subdomain, Table S2: Changes to the wording of items across the testing rounds for the final set of 20 items in the ECDI2030.
