# Supplementary material for: Identifying and Minimizing Errors in the Measurement of Early Childhood Development: Lessons Learned from the Cognitive Testing of the ECDI2030
Source: Int J Environ Res Public Health. 2021 Nov 20;18(22):12181. doi: 10.3390/ijerph182212181 (PMC8618056; doi:10.3390/ijerph182212181)
Supplement: Supplementary file 1 [file ijerph-18-12181-s001.zip › ijerph-1410058-supplementary.pdf]

Table S1: Questions used for cognitive testing, by domain and subdomain

Item	Domain	Subdomain
Can (name) identify at least 10 letters of the alphabet?	Learning	Literacy
Can (name) read at least four simple, popular words?	Learning	Literacy
If you show (name) two objects or people of different size, can he/she tell you which one is the big one and which is the small one?	Learning	Numeracy
Can (name) count up to five objects, for example, five fingers or five blocks?	Learning	Numeracy
If you ask (name) to give you three objects, such as three stones or three beans, does the child give you the correct amount?	Learning	Numeracy
Can (name) count 10 objects, for example, 10 fingers or 10 blocks?	Learning	Numeracy
Can (name) identify all written numbers from 1 to 5?	Learning	Numeracy
Does (name) know that a goat weighs more than a mouse?	Learning	Numeracy
If you show (name) an object he/she knows well such as a cup or animal, can he/she consistently name it?	Learning	Expressive language
Can (name) say ten or more separate words such as names like "Mama" or objects like "ball"?	Learning	Expressive language
Can (name) correctly ask questions using any of the words "what," "which," "where," or "who"?	Learning	Expressive language
Can (name) explain in words what common objects like a cup or chair are used for?	Learning	Expressive language
Can (name) speak using sentences of three or more words that go together, for example, "I want water" or "The house is big"?	Learning	Expressive language
Can (name) sing a short song or repeat parts of a rhyme from memory by him/herself?	Learning	Expressive language
Can (name) correctly use any of the words "I," "you," "she," or "he," for example, "I go to the store," or "He eats rice"?	Learning	Expressive language

Can (name) correctly use the words "on," "in," or "under" to describe where an object is, for example, "The cup is on the table" instead of "The cup is in the table"?	Learning	Expressive language
Does (name) ask "why" questions such as, "Why are you tall?"?	Learning	Expressive language
Can (name) talk about things that have happened in the past using correct language, for example, "Yesterday I played with my friend" or "Last week she went to the market"?	Learning	Expressive language
When looking at pictures or watching others, can (name) tell you what action is taking place if you ask (name)?	Learning	Expressive language
Can (name) write their own name?	Learning	Pre-writing
Does (name) take an object and pretend it is something else?	Learning	Approaches to learning
Compared with children of the same age, does (name) have difficulty learning things?	Learning	Approaches to learning
Can (name) identify at least one color, like red, blue, or yellow?	Learning	General knowledge
Can (name) name shapes like circles, triangles and squares?	Learning	General knowledge
When a familiar adult asks (name) to do something, does (he/she) comply?	Learning	Receptive language
When you say “no” or try to get (name) to stop doing something, does (he/she) respond immediately and hesitate or stop what they are doing?	Learning	Receptive language & executive functioning
Does (name) follow complex directions with more than one step? For example, “Go to the kitchen and bring me a spoon” or "Get me your cup and put water in it"?	Learning	Receptive language & executive functioning
Does (name) correctly follow simple directions on how to do something?	Learning	Receptive language & executive functioning
Can (name) easily switch back and forth between activities like going back to a game or playing with a toy after being interrupted?	Learning	Executive functioning
Does (name) usually finish an activity he/she enjoys like playing a game or looking at a book?	Learning	Executive functioning
When given something to do, is (name) able to do it independently?	Learning	Executive functioning
Can (name) imitate a circle?	Health	Fine motor development

Can (name) imitate a straight line?	Health	Fine motor development
Can (name) pick up a small object with two fingers, like a stick or a rock from the ground?	Health	Fine motor development
Can (name) dress him/herself, that is, putting on pants and shirt without help?	Health	Self-care & gross motor development
How often does (name) go to the bathroom alone without the help of an adult?	Health	Self-care
Can (name) jump with both feet leaving the ground?	Health	Gross motor development
Can (name) throw the ball on purpose by extending an arm, and not just dropping the ball?	Health	Gross motor development
If someone gently throws a ball to (name), can (name) catch it, and hold on to it, for at least a few seconds?	Health	Gross motor development
Can (name) walk well, with coordination?	Health	Gross motor development
Does (name) run more than a few steps, with feet lifted up, without falling or bumping into objects?	Health	Gross motor development
Does (name) ask about familiar people other than parents when they are not there, for example, "Where is Grandma?")?	Psychosocial well-being	Social cognition
Can (name) say what others like or dislike, for example, "Mama doesn't like fruit" or "Papa likes football"?	Psychosocial well-being	Theory of mind
Compared with children of the same age, does (name) have difficulty playing?	Psychosocial well-being	Social competence
Does (name) get along well with other children?	Psychosocial well-being	Social competence
Does (name) share things with other children or family members without being told to do so?	Psychosocial well-being	Social competence
Thinking about the past 6 months, would you say that (name) has demonstrated respect for adults?	Psychosocial well-being	Social competence
How often, does (name) like to meet new people?	Psychosocial well-being	Social competence
Does (name) have difficulty taking turns when playing together with others?	Psychosocial well-being	Social competence
If (name) has a problem with another child, fighting over a toy or something, does your child come to you or another adult and ask for help?	Psychosocial well-being	Social competence

How often does (name) help with some things in the house when asked?	Psychosocial well-being	Empathy
How often, does (name) notice if a person is crying?	Psychosocial well-being	Empathy
Does (name) worry when a known or familiar person is sick or hurt?	Psychosocial well-being	Empathy
Does (name) offer to help someone who seems to need help?	Psychosocial well-being	Prosocial behaviour
Does (name) settle down after periods of exciting activity?	Psychosocial well-being	Emotional development
When (name) needs to use the toilet, does she/he show you by pulling on their clothes, holding herself/himself, crying, or some other way?	Psychosocial well-being	Self-regulation
Does (name) get distracted easily?	Psychosocial well-being	Self-regulation
Does (name) often kick, bite, or hit other children or adults?	Psychosocial well-being	Externalizing behaviour
Does (name) destroy things that s/he shouldn't destroy?	Psychosocial well-being	Externalizing behaviour
Thinking about the past 6 months, how often did (name) seem to be unhappy, sad or depressed?	Psychosocial well-being	Internalizing behaviour
Does (name) become extremely withdrawn or shy in new situations?	Psychosocial well-being	Internalizing behaviour

Table S2: Changes to the wording of items across the testing rounds for the final set of 20 items in the ECDI2030

	Domain - Subdomain	Original item	Item Description	Item Used in Round One (India and Jamaica)*	Items Used in Round Two (USA)*	Items Used in Round Three (Mexico and Bulgaria)*	Items Used in Round Four (Uganda)*	Items Used in Round Five (Mexico)*	Final Revision	Revisions Integrated	Final ECDI2030 Item
1	**Health - Gross motor development**	Can the child walk on an uneven surface (e.g., a bumpy or steep road) without falling?	The child can walk more than several steps up and down an incline (e.g., a hill or a ramp) without falling, or on a bumpy surface (e.g., gravel) without falling.	Not used	Can (name) walk well, with coordination?	**Can (name) walk on an uneven surface (e.g. a bumpy or steep road) without falling?**	Same	Can (name) walk on an uneven surface, **for example**, a bumpy or steep road, without falling?	Same	R2-3: Replaced with new item applicable for older children<br><br>Field Test: Editorial changes to improve administration	*Can (name) walk on an uneven surface, for example, a bumpy or steep road, without falling?*

2	**Health – Gross motor development**	Can the child jump with both feet leaving the ground?	The child can jump such that both feet are off the ground at the same time. The child can jump straight up, or in any direction (forward, backward, to the side).	Not used	Can (name) jump with both feet leaving the ground?	Same	Same	Can (name) jump **up** with both feet leaving the ground?	Same	Field Test: Revision to match item description	*Can (name) jump up with both feet leaving the ground?*
3	**Health – Self-care and gross motor development**	Can the child dress him/herself (e.g., put on his/her pants and shirt without help)?	The child can put on his/her own clothes (e.g., pants, shirt, dress, shoes) correctly (e.g., shirt not backwards) and without help. The child does not need to be able to tie the shoes or to close complex fixtures (e.g., buttons, zippers, clasps). This item targets gross motor development.	Not used	Can (name) dress him/herself, that is, putting on pants and shirt without help?	Can (name) **correctly** dress him/herself, that is, putting on pants and shirt without help?	Same	Can (name) dress him/herself, that is, put on pants and shirt without help?	Same	R2-3: Added 'correctly' for added clarity of intent of the question<br><br>Field Test: Word 'correctly' removed due to focus on gross motor skills	*Can (name) dress him/herself, that is, put on pants and shirt without help?*
4	**Health – Fine motor development**	Can the child fasten and unfasten buttons without help?	The child is able to button and unbutton shirts, pants, or other pieces of clothing without the assistance of an adult or other child.	Not used	Not used	Not used	Not used	Can the child fasten and unfasten buttons without help?	Can **(name)** fasten and unfasten buttons without help?	Final: Editorial changes to improve administrati on	*Can (name) fasten and unfasten buttons without help?*
5	**Learning – Expressive language**	Can the child say ten or more separate words (e.g., names like "Mama" or	The child can clearly say ten or more separate words. These words	Not used	US: Can (name) say ten or more separate words such as names	Same	Same	Can (name) say **at least** ten or more words like "Mama" or "ball"?	Can (name) say 10 or more words	Field Test: Revised to simplify language, shorten	*Can (name) say 10 or more words like "mama" or "ball"?*

		objects like "ball")?	can be real words (e.g., objects, names) or "made up" words that the child consistently uses to convey meaning (e.g., a nickname for a person or food).		Like "Mama" or objects like "ball"?				like "mama" or "ball"?	question, and remove the qualifier 'separate' as it was not necessary for intent of question<br><br>Final: Removed 'at least'	
**6/7**	**Learning – Expressive language**	Can the child speak using sentences of three or more words that go together (e.g., "I want water" or "The house is big")?	The child can clearly speak by forming short sentences of three/five or more words. These Sentences should reflect children's ability to link words to convey complex thoughts. For example, the sentence could include a subject, verb, and object combination (e.g., "I see a dog"), or it could include a directive (e.g., "I want more"). Simple word combinations that do not convey complex meaning (e.g., "go, go, go") do not count as sentences.	Not used	Can (name) speak using sentences of three or more words that go together, for example, "I want water" or "The house is big"?<br><br>Can (name) speak using sentences of 5 or more words that go together, for example "The house is very big"?	Same	Same	Same	Same	None	*Can (name) speak using sentences of three or more words that go together, for example, "I want water" or "The house is big"?*<br><br>*Can (name) speak using sentences of 5 or more words that go together, for example "The house is very big"?*

**8**	**Learning – Expressive language**	Can the child correctly use any of the words "I," "you," "she," or "he" (e.g., "I go to store," or "He eats rice")?	The child can use any pronouns (e.g., I, you, he, she, we, they) correctly in sentences.	Not used	Can (name) correctly use any of the words "I," "you," "she," Or "he," for example, "I go to the store," or "He eats rice"?	Same	Same	Same	Can (name) correctly use any of the words "I," "you," "she," or "he," for example **"I want water,"** or "He eats rice"?	Final: Example changed because "go to store" may not be relevant in all contexts	*Can (name) correctly use any of the words "I," "you," "she," or "he," for example "I want water," or "He eats rice"?*
**9**	**Learning – Expressive language**	If you show the child an object he/she knows well (e.g., a cup or animal), can he/she consistently name it?	When the child is shown a familiar object, the child uses the same word to refer to the same object, even if the word used is not fully correct.	Not used	If you show (name) an object he/she knows well such as a cup or animal, can he/she consistently name it?	Same	Same	Same	If you show (name) an object (he/she) knows well, such as a cup or animal, can (he/she) consistently name it?<br><br>**By consistently we mean that (he/she) uses the same word to refer to the same object,**	Final: The meaning of 'consistently' was not universally understood and was often confused with 'correctly,' thus a definition was included as a way to clarify the difference.	*If you show (name) an object (he/she) knows well, such as a cup or animal, can (he/she) consistently name it?*<br><br>*By consistently we mean that (he/she) uses the same word to refer to the same object, even if the word used is not fully correct.*

									**even if the word used is not fully correct.**		
10	**Learning - Literacy**	Can (name) identify or name at least 10 letters of the alphabet?	To identify or use the name of at least 3 (or 5 or 10) letters of the local alphabet. Knowledge of letters could be shown if a child is able say the alphabet, if a child can correctly say what letters are in his/her name, if a child can read a letter's symbol, or if a child can point to a letter when Asked. This item may be culturally related, for example in contexts in which languages do not have letters (e.g., China).	Can (name) identify or name at least 10 letters of the alphabet?	Can (name) identify at least 10 letters of the alphabet?	Can (name) identify at least **3** letters of the alphabet?<br><br>If yes, can (name) identify at least 5 letters of the alphabet?<br><br>If yes, can (name) identify at least 10 letters of the alphabet?	Same	Can (name) recognize at least **3** letters of the alphabet?<br><br>If yes, can (name) recognize at least 5 letters of the alphabet?<br><br>If yes, can (name) recognize at least 10 letters of the alphabet?	Can (*name*) recognize at least **5** letters of the alphabet?	R2-3: To capture the variation of abilities in the item description<br><br>Field Test: To more directly apply to nonverbal children<br><br>Final: Increased number to 5	*Can (name) recognize at least 5 letters of the alphabet?*
11	**Learning – Pre-writing**	Can (name) write their own name?	The child can use the correct letters of the local alphabet in the proper order to spell out his/her own name in print/block letters (not cursive/handwriting). The	Not used	Can (name) write their own name?	Can (name) **correctly** write **his/her** own name?	Same	Can (name) write **his or her** own name?	Can (*name*) write **(*his/her*)** own name?	R1: Editorial change for subject/verb agreement<br>R2-3: Included qualifier that name should be written correctly	*Can (name) write (his/her) own name?*

			child need not be able to write both first and last name and writing a nickname would be sufficient.							Field Test: Word 'correctly' removed due to focus on fine motor skills<br><br>Final: Editorial changes to improve administration	
**12**	**Learning - Numeracy**	Does (name) know the name and recognize the symbol of all numbers from 1 to 10?	The child can clearly identify or recognize all written numbers from 1 to 5 in his/her native language. The child could demonstrate knowledge of written numbers if he/she could say the number when shown the symbol written on paper (e.g., says "one" when shown the number "1") or if a child can point to a number when asked (e.g., which is the number "1"?). This item may be culturally dependent..	Does (name) know the name and recognize the symbol of all numbers from 1 to 10?	US: Can (name) identify all written numbers from 1 to 5?	Same	Can (name) **recognize** all numbers from 1 to 5?	Does (name) **know** all numbers from 1<br>to 5?	Can (name) **recognize** all numbers from 1 to 5?	R3: Changed to 'recognize' because 'identify' was a difficult term for lower educated mothers; Deleted 'written' to simplify and avoid confusion about numbers versus symbols (for example, number "2" versus word "two")<br><br>R4: Word "know" was tested as a more colloquial alternative<br><br>Field test: question	*Can (name) recognize all numbers from 1 to 5?*

										changed back to “recognize” because “know” was falsely interpreted as “knowing how to count” by some mothers.	
**13**	**Learning - Numeracy**	If you ask the child to give you three objects (e.g., stones, beans), does the child give you the correct amount?	The child can hand or bring the respondent a specific and correct number of items that were requested by the respondent. The child does not give too many or too few objects.	Not used	If you ask (name) to give you three objects, such as three stones or three beans, does the child give you the correct amount?	Same	Same	Same	If you ask (*name*) to give you 3 objects, such as 3 stones or 3 beans, does **(*he/she*)** give you the correct amount?	Final: Editorial change to improve administration	*If you ask (name) to give you 3 objects, such as 3 stones or 3 beans, does (he/she) give you the correct amount?*
**14**	**Learning - Numeracy**	Can the child count up to five objects (e.g., fingers, people)?	The child can clearly and correctly count a finite number of at least 10 objects. If the child skips numbers in the sequence or says numbers out of order, the response should be No.	Not used	Can (name) count 10 objects, for example, 10 fingers or 10 blocks?	Can (name) count 10 objects, for example, 10 fingers or 10 blocks **correctly and in order without skipping numbers?**	Same	Can (name) count 10 objects, for example, 10 fingers or blocks, **without mistakes?**	Same	R2-3: To clarify the purpose of the item (i.e. “count objects correctly”)<br><br>Field Test: Changed wording to simplify language and make consistent with other items that ask about counting	*Can (name) count 10 objects, for example, 10 fingers or blocks, without mistakes?*

**15**	**Learning – Executive functioning**	When given something to do, is (name) able to do it independently?	The child can do an activity on his or her own for an appropriate length of time (e.g., coloring or playing with toys) without constantly asking for assistance from someone else or giving up too quickly. This item targets sustained attention, persistence, and ability to work independently.	When given something to do, is (name) able to do it independently?	When given something to do, is (name) able to do it independently?	Same	**Can (name) do an activity such as coloring or something else** independently?	Can (name) do an activity such as coloring **without repeatedly asking for help or giving up too quickly?**	Can (*name*) do an activity, such as colourin g **or playing with building blocks**, without repeatedl y asking for help or giving up too quickly?	R3: Changed wording to convey activities as opposed to chores and provided an example<br><br>Field Test: Revised wording to remove 'independe ntly' because it was not consistently understood<br><br>Final: Added another example	*Can (name) do an activity, such as colouring or playing with building blocks, without repeatedly asking for help or giving up too quickly?*
**16**	**Psychosocial wellbeing – Social cognition**	Does the child ask about familiar people other than parents when they are not there (e.g., "Where is the neighbor?")?	The child asks questions about other people he/she knows, other than a parent or primary caregiver (e.g., friend, sibling) when that person is absent or not in sight. This can include asking about where people are, when they will visit, or what they are doing. This item targets social cognition skills and expressive language	Not used	Does (name) ask about familiar people other than parents when they are not there, for example, "Where is Grandma?"?	Same	Same	Same	Same	None	*Does (name) ask about familiar people other than parents when they are not there, for example, "Where is Grandma?"?*

17	**Psychosocial wellbeing – Prosocial behaviour**	Does (name) offer to help someone who seems to need help?	The child offers to help either adults or other children (including siblings) when they seem to need help, without being told to do so.	Not used	Does (name) offer to help someone who seems to need help?	Same	Same	Same	Same	None	*Does (name) offer to help someone who seems to need help?*
18	**Psychosocial wellbeing – Social competence**	Does (name) get along well with other children?	The child usually (more often than not) can interact or play with other children in a positive Manner. This item targets early social skills.	Does (name) get along well with other children?	Does (name) get along well with other children?	Same	Same	Same	Same	None	*Does (name) get along well with other children?*
19	**Psychosocial wellbeing – Internalizing behaviour**	Thinking about the past 6 months, how often did (name) seem to be unhappy, sad or depressed?	The child could express unhappiness, sadness, or depression by appearing withdrawn, Unenthusiastic, or tearful, for example. These emotions could also be communicated through verbal expression of sadness. This question is distinct from the child simply being tired though it may look Similar. This item targets emotional well-being and mental health.	Not used	Thinking about the past 6 months, how often did (name) seem to be unhappy, sad or depressed?<br><br>Almost never, sometimes, many times, almost always, don't know<br><br>Often or very true; Sometimes or somewhat true; Never or not true; Don't know	Same	Thinking about the past 6 months, how often did (name) seem to be very unhappy, sad or depressed?	How often does (*name*) seem to be very sad or depressed?<br><br>**Would you say: daily, weekly, monthly, a few times a year or never?**	Same	R3: Included a qualifier of 'very' to detect emotions that were extreme and avoid reports of typical unhappines s due to everyday events<br><br>Field Test: Deleted unhappy because it is conceptuall y the same as sad and changed response options to include a	*How often does (name) seem to be very sad or depressed?*<br><br>*Would you say: daily, weekly, monthly, a few times a year or never?*

										shorter reference period that has a lower respondent burden	
**20**	**Psychosocial Wellbeing – Externalizing behaviour**	Does (name) kick, bite, or hit other children or adults?	The child frequently (more than once per week) shows aggression toward other children (other than siblings) or adults through kicking, biting, hitting, slapping, or unwanted tackling. This item Targets early aggressive or externalizing behavior problems.	Does (name) kick, bite, or hit other children or adults?	Does (name) often kick, bite, or hit other children or adults?	Same	Same	**Compared with children of the same age, how much** does (*name*) kick, bite, or hit other children or adults?<br><br>**Would you say: not at all, the same or less, more or a lot more?**	Same	Field Test: Changes include a frequency scale and reference to other children	*Compared with children of the same age, how much does (name) kick, bite, or hit other children or adults?*<br><br>*Would you say: not at all, the same or less, more or a lot more?*

Response Options: Yes/No/Don't Know, except where indicated
***Some revisions noted by bolding**
